# A Review of Edible Jujube, the *Ziziphus jujuba* Fruit: A Heath Food Supplement for Anemia Prevalence

**DOI:** 10.3389/fphar.2020.593655

**Published:** 2020-11-26

**Authors:** Jianping Chen, Karl W. K. Tsim

**Affiliations:** ^1^ Shenzhen Key Laboratory of Hospital Chinese Medicine Preparation, Shenzhen Traditional Chinese Medicine Hospital, The Fourth Clinical Medical College of Guangzhou University of Chinese Medicine, Shenzhen, China; ^2^ Division of Life Science and Center for Chinese Medicine, The Hong Kong University of Science and Technology, Hong Kong, China

**Keywords:** *Ziziphus jujuba*, Rhamnaceae, blood deficiency, bio-active ingredient, food supplement

## Abstract

The fruits of *Ziziphus jujuba*, commonly known as jujube, red date or Chinese date, are taken as fresh or dried food, and as traditional medicine worldwide due to high nutritional and health values. Traditionally in China, jujube is considered as a medicinal fruit that is being used in treating blood deficiency. In this review, the beneficial effects of jujubes on the hematopoietic functions are summarized and discussed. As illustrated in cell and animal models, the application of jujube extract possessed beneficial effects, including regulation of erythropoiesis via activation of hypoxia inducible factor-induced erythropoietin, potential capacity in recycling heme iron during erythrophagocytosis and bi-directional regulation of immune response. Thus, the blood-nourishing function of jujube is being proposed here. Flavonoid, polysaccharide and triterpenoid within jujube could serve as the potential active ingredients accounting for the aforementioned health benefits. Taken together, these findings provide several lines of evidence for further development of jujube as supplementary products for prevention and/or treatment of anemia.

## Introduction

**FIGURE 1:**
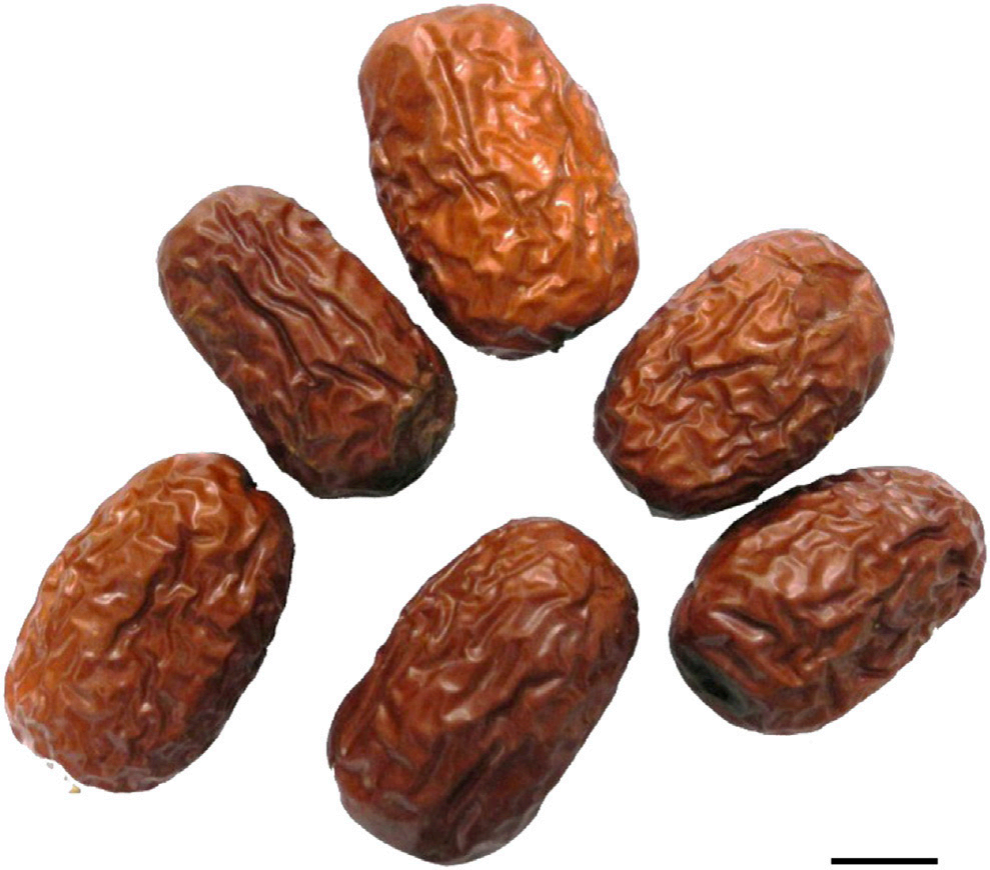
The photo of *Z. jujuba* fruits. The photo of dried jujubes collected from Xinjiang province, one of major production regions of jujubes in China. Bar: 1 cm.

Jujube is usually called red date or Chinese date, which is the fruit of *Ziziphus jujuba* Mill. that belongs to Rhamnaceae family. Jujube is native to China, and which has been commonly consumed as food supplement and traditional Chinese medicine (TCM) for thousands of years (Figure 1). Today, jujube plant is distributed widely not only in China but also in other countries, e.g. Korea, India, Japan, Europe and the United States. In *Huangdi Neijing* (475–221 BC), a classic medical text from ancient China, jujube was recorded as one of extremely valuable fruits. According to *Shennong Bencao Jing* written between 300 BC and 200 AD, one of the earliest books specializing in Chinese medicine, jujube was regarded as one of the top-grade medicinal herbs that could extend one’s life expectancy by nourishing blood, increasing sleep quality and improving digestive system. Along with growing number of studies on jujube, various beneficial nutrients within jujube are being proposed, including carbohydrate, mineral, vitamin, sugar and amino acid. Thus, jujube is considered as a popular nutritious food, worldwide (Li et al., 2007; San and Yildirim, 2010; USDA, 2012; Guo et al., 2013; Reche et al., 2019). Being a Chinese herb or health food supplement, recent studies have indicated that jujube possesses a wide range of pharmacological activities in nervous system, cardiovascular system, as well as anti-oxidation and anti-cancer properties (Table 1).

**TABLE 1:** Health beneficial properties of jujube.

Biological functions	Findings	References
Nervous system	Oleamide isolated from jujube at 14–16 mg/kg significantly restored memory and/or cognitive impairment in mice induced by scopolamine	Heo et al., 2003
	Jujube hydroalcoholic extract at100, 250, 500, and 1,000 mg/kg ameliorated seizures, oxidative stress, and cognitive impairment in epilepsy rat model	Pahuja et al., 2011
	Jujube at 0.72, 1.8, and 4.5 g/kg improved learning and memory ability in ovariectomized rat model in the Morris water maze experiment	Li et al., 2013
Cardiovascular system	The seeds of Z. jujuba (30, 100, or 300 mg/kg) and its active component jujuboside B (10, 30, or 100 mg/kg) were reported to exhibit anti-platelet aggregation activity	Seo et al., 2013
	Jujube extracts at 25 and 50 µg/ml suppressed lipid accumulation and GPDH^a^ activity in 3T3-L1 preadipocytes	Kubota et al., 2009
	Consumption of jujube infusion (10 g/100 ml) three times/day prior to main meals for 12 weeks in patients with type 2 diabetes mellitus showed a robust improvement in lipid profiles and glycaemic index	Yazdanpanah et al., 2017
Anti-oxidative activity	Jujube extracts at various concentrations (0.25–1.0 mg/ml) inhibited DPPH radical, and its DPPH radical scavenging effect was in a dose dependent manner	Li et al., 2005
	Jujube extracts (0–3.0 mg/ml) protected tBHP-induced oxidation insult on PC12 cells via activation of ARE-mediated transcriptional activity^b^	Chen et al., 2013
	Jujube polysaccharides (0–3.0 mg/ml) possessed the potential effect to scavenge hydroxyl radicals, and the scavenging rates increased dose-dependently	Ji et al., 2020
Anti-cancer activity	Dietary jujube for 70 days (5% or 10% w/w) inhibited tumor progression and promoted the tumor apoptosis in CAC cancer mice	Periasamy et al., 2020
	Jujube extracts (0–200 µg/ml) induced dose-dependently effect on apoptosis and a differential cell cycle arrest, i.e., G1 and G2/M arrest in HepG2 cells	Huang et al., 2007
	3OTPCA at 5–80 µM, a triterpenoid isolated from jujube induces apoptotic cell death in human leukemia cells via the generation of reactive oxygen species and activation of UPR^c^	Mitsuhashi et al., 2017
Other medicinal properties	Mice given with 1 and 10% of Z. jujuba essential oil at different concentrations (0.1, 1, and 10%) induced a greater activity on the length of hair	Yoon et al., 2010
	Glucans (10, 20, 50, and 100 µg/ml) from jujube possessed effect on regeneration of damaged skin through prompting cellular survival and cell migration	Fazio et al., 2020
	Jujube extracts at various concentrations (100–400 mg/kg) showed potent anti-asthmatic activity in ovalbumin (OVA) -induced allergic asthma of mice	Ninave and Patil, 2019

a GPDH, glycerol-3-phosphate dehydrogenase.

b tBHP, tert-butyl hydroperoxide; ARE, Anti-oxidant response element.

c CAC, colitis-associated colon; UPR, unfolded protein response.

Clinically, blood deficiency is usually encountered in women due to the loss of menstrual blood, or in patient who has lost blood or suffered from chronic malnutrition. Jujube is a functional food, which is believed to possess robust effect in tonifying the blood, in order to prevent blood deficiency in human. According to the theory of TCM, blood deficiency shows similarity to anemia of individual in western medicine (Shi et al., 2019). In line to this notion, pharmacological studies have reported that jujube has potential hematopoietic functions both *in vivo* and *in vitro* (Xu et al., 2004; Chen et al., 2014b). Specific targets supporting the clinical usage of jujube in hematopoietic functions however remain unclear. Here, we are focusing on a discussion of jujube associating with hematopoietic functions, i.e., erythropoiesis, erythrophagocytosis and immune functions (Table 2). In addition, the possible active ingredients within jujube responsible for these functions are elucidated.

**TABLE 2:** Hematopoietic properties of jujube.

Findings	Model	Treatment	References
Jujube extract exhibited anti-platelet aggregations effect in a dose dependent manner	Platelet-rich plasma was prepared from SD rats; *in vitro* platelet aggregation study	Pretreatment with jujube extracts at 30, 100, 300 mg/ml for 5 min at 37°C; collagen (2 mg/ml)-, thrombin (0.4 U/ml)-, and AA (100 mM) was employed to induce PLT aggregations^a^	Seo et al., 2013
Dietary jujube increased RBC, Hb, and HCT levels in cancer mice	Mice with CAC ^b^ were induced by injecting with azoxymethane followed by three cycles of 2% (w/v) DSS; hematological examination	Mice were given with Z. jujuba fruit for 70 days (5 or 10%°w/w)	Periasamy et al., 2020
Jujube water extract stimulated EPO expression via hypoxia inducible factor signaling pathway	Cultured Hep3B cells; mRNA and protein expression	Treatment with jujube extracts at different dosages (0.75–3.0 mg/ml) for 24 h	Chen et al., 2014b
Jujube induced expressions of iron recycling enzymes via Nrf2/ARE pathway	Cultured RAW 264.7 macrophages; mRNA expression; ARE transcriptional activity	Jujube water extract at 0.375, 0.75, 1.5 and 3.0 mg/ml. cells were treated for 24 h	Chen J. et al., 2016
Jujube water extract corrected anemia in iron deficiency rats	Iron-deficient diet to induce anemic rats; hematological analysis	jujube extracts at various concentrations (2.7, 5.4, and 10.8 g/kg/day); rats was treated for 14°weeks	Yang et al., 2016
Jujube extract stimulated thymus and spleen indices to enhance nonspecific immunity of mice model	Kunming mice; relative thymus and spleen weight	Oral administration of jujube extracts (50, 150, and 250 mg/kg/day) for 4°weeks	Li et al., 2011
Jujube extract showed anti-inflammatory effect via inhibition of nitric oxide expression	Chronic inflammatory rat model was induced by interscapular implantation of a sterile cotton pellet (50 mg); nitrite/nitrate estimation	The hydroalcoholic extract of jujube at 200 and 400 mg/kg was given to rats for 7°days	Goyal et al., 2011
Jujube extract regulated pro-inflammatory cytokine expressions under different conditions via NF-kB signaling	Cultured RAW 264.7 cells; mRNA and protein expression; luciferase activity	Jujube extract at different dosages (0–3.0 mg/ml) for 24 h	Chen et al., 2014a
Ju-B-2 from jujube induced spleen cells proliferation, and the structural-activity relationship of which in stimulating immune response were proposed	Cultured spleen cells obtained from Balb/c male mice; immunomodulating activity	Ju-B-2, Ju-B-2-spl, Ju-B-3 at 10, 30, 100 µg/ml; LPS at 2.5 µg/ml served as positive control; cells were treated for 3 days	Zhao et al., 2006

a AA, arachidonic acid; PLT, platelet.

b DSS, dextran sulfate sodium; CAC, colitis-associated colon cancer.

### Potential Bio-Active Ingredients of Jujube in Hematopoietic Function

**FIGURE 2:**
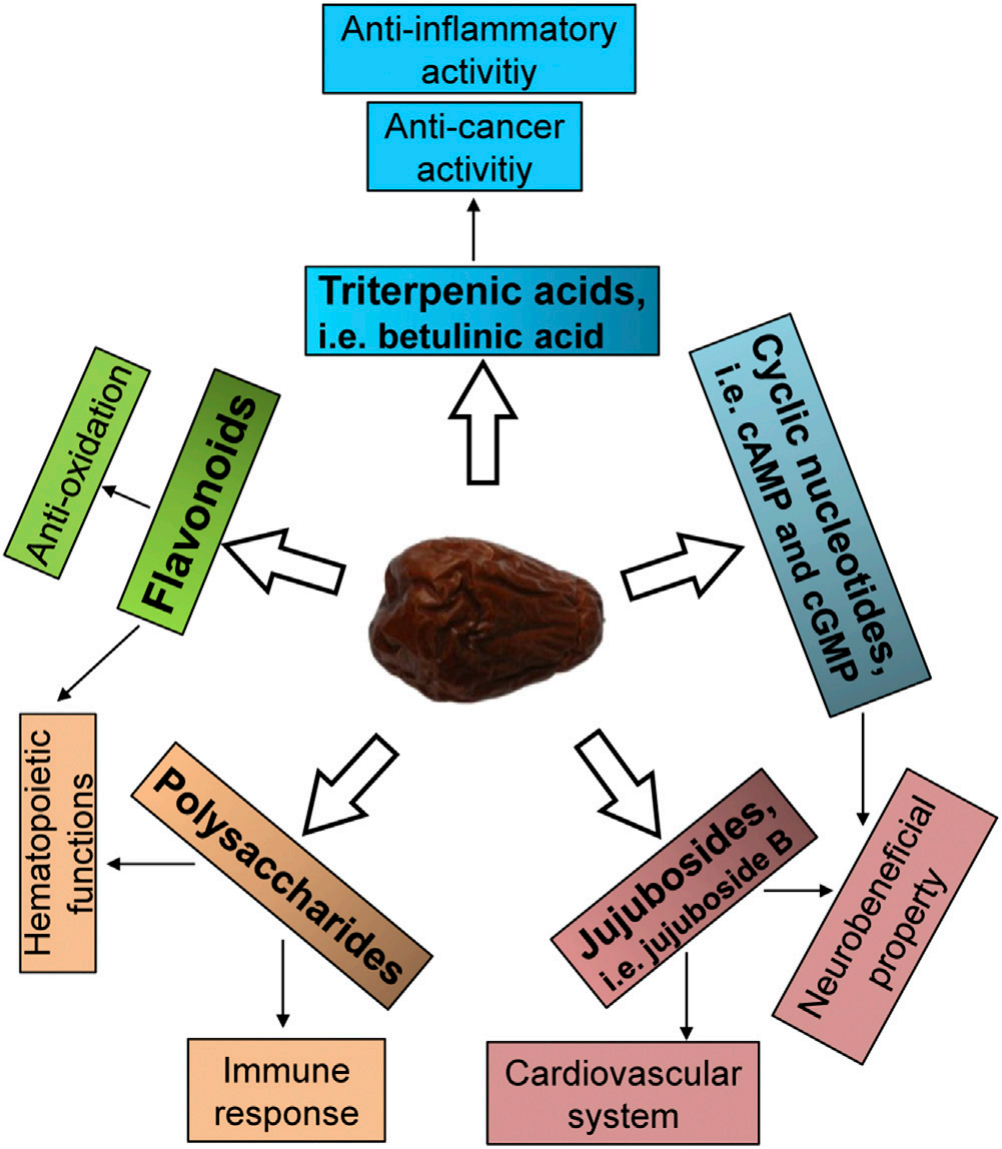
Active ingredients of jujube. The potential active ingredients relating to the activities of jujube are summarized.

**FIGURE 3:**
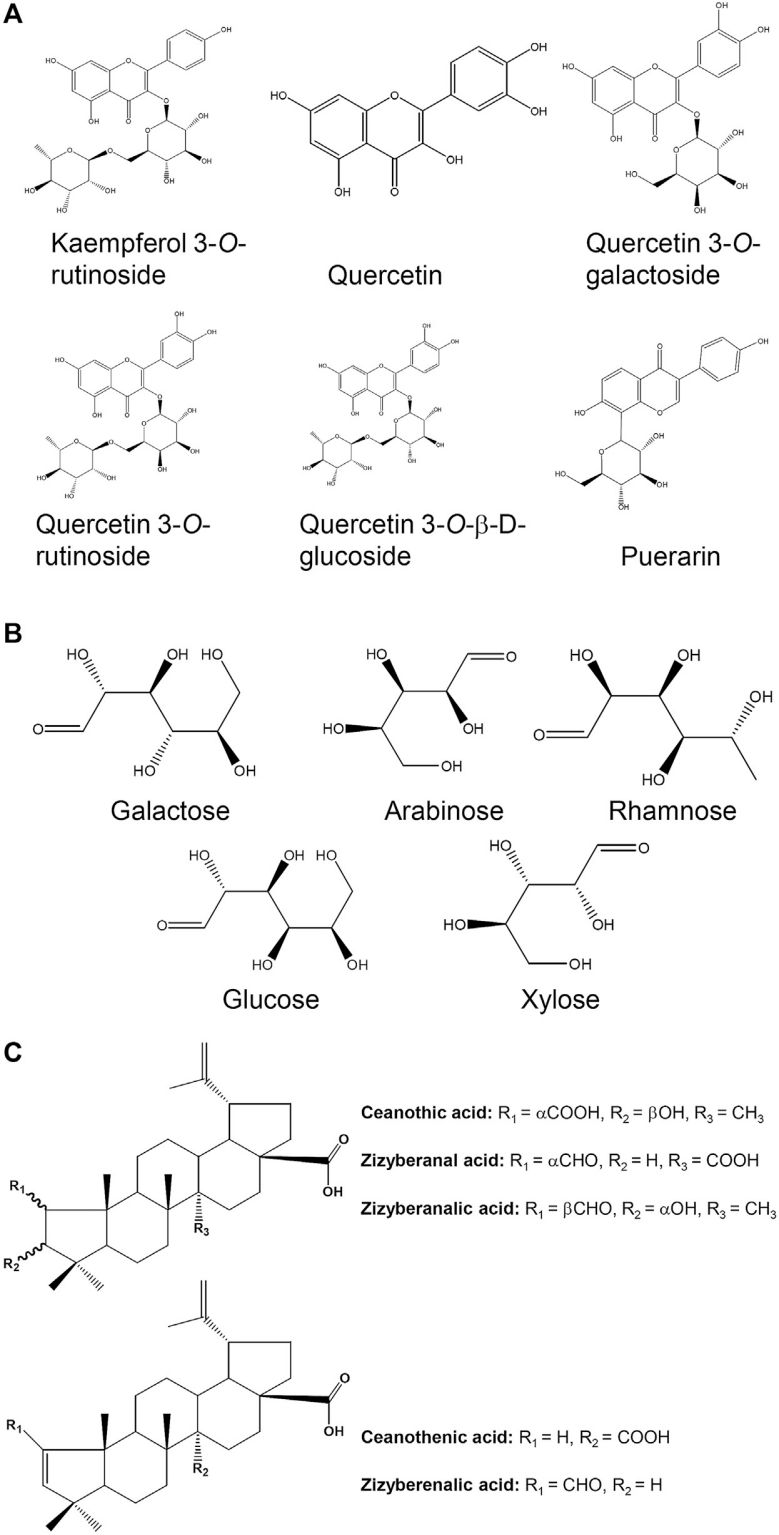
Chemical structures for compounds in jujube possessing potential hematopoietic activity. **(A)** The chemical structures of six flavonoids found in the fruits. **(B)** The composition in monosaccharide purified from jujube. **(C)** The chemical structures of varieties of triterpenic acids found in the fruit.

Jujube has a promising source of flavonoids, polysaccharides, terpenoids, saponins, nucleotides and others (Figure 2). Here, the trophic ingredients having potential beneficial effects on hematopoietic function are highlighted. At present, a variety of flavonoids were isolated and identified in jujube (Cheng et al., 2000; Pawlowska et al., 2009; Choi et al., 2011; Gao et al., 2013). Flavonoids from jujube have been found to stimulate the expression of erythropoietin (EPO), a hormone stimulating blood production (Zheng et al., 2011), and therefore we speculated that jujube flavonoid might be one of the active compounds that possessed the ability to induce the expression of EPO. Supporting this notion in the pHRE-Luc (the DNA promoter construct of *EPO* gene, hypoxia response element) transfected cultured HEKT293T cells, the application of kaempferol at 10 μM for 24 h could significantly induce the transcriptional activity of pHRE-Luc with 127% of increase, as compared to control group (Xu et al., 2018). Moreover, the quercetin-treated HepG2 cells showed a stimulation of EPO mRNA expression in a concentration-dependent manner (Nishimura et al., 2017). Similarly, the protein level of HIF-1α was markedly up regulated at the treatment of 10 μM quercetin (Nishimura et al., 2017). Indeed, kaempferol and quercetin derivatives, including kaempferol 3-O-rutinoside, quercetin, quercetin 3-O-rutinoside, quercetin 3-O-galactoside and quercetin 3-O-β-D-glucoside (Figures 3A), were identified in jujube (Gao et al., 2012; Chen et al., 2013). Therefore, jujube flavonoid can induce EPO expression, probably, through HIF-α protein accumulation. In addition, the combination of catalpol and puerarin with doses of 65.4 and 32.7 mg/kg, respectively, enhanced the expressions of EPO and EPO receptor in ischemic/reperfusion rats (Xue et al., 2016). In parallel, puerarin was identified in the seeds of jujube (Cheng et al., 2000). Flavonoids are common chemical presented in a wide range of plants. Plant extracts rich in aforesaid flavonoids are considered, therefore, potentially useful as therapeutic agents for anemia.

Polysaccharides from plant have been demonstrated to possess various bio-activities, e.g., anti-oxidation, anti-complementary and immunological activities (Li et al., 2003; Gao et al., 2007; Chen M. et al., 2016). Several polysaccharides have been isolated and purified from the jujube. The polysaccharides extracted from jujube usually consist of five monosaccharides, i.e., galactose, arabinose, rhamnose, glucose and xylose (Zhao et al., 2008) (Figures 3B). Fractions named as ZSP1, ZSP2, ZSP3 and ZSP4 with weight ratio of 29.3:17.6:37.2:15.9 have been purified from jujube (Li et al., 2011). The fractions of ZSP3 and ZSP4 at various concentrations (30–200 μg/ml) were applied onto peritoneal macrophages, and the cell proliferation was detected by MTT assay. These two fractions were found to dose-dependently induce proliferation of spleen lymphocyte, having the highest response under the treatment of jujube polysaccharide at 200 μg/ml. This finding suggests the immunological activity of jujube polysaccharide. In line with this, Ju-B-2, a molecular weight of over 2,000 kDa polysaccharide from jujube, was shown to have the immune activity. Application of Ju-B-2 at 10–100 μg/ml onto cultured spleen cells for 3 days induced cell proliferation. Furthermore, the authors proposed the structures of rhamnogalacturonan and its side chains of Ju-B-2 polysaccharide contributing to the immune response (Zhao et al., 2006). Although several reports support the beneficial effects of jujube polysaccharides in preventing anemia, its detail action mechanisms are still rather limit. Hence, possible signaling pathways involved in jujube polysaccharide-treated *in vitro* or *in vivo* models are needed for further investigation.

Triterpenic acids have been isolated and purified from jujube, including ceanothenic acid, zizyberanal acid, zizyberenalic acid, zizyberanalic acid, and ceanothic acid (Figures 3C) (Yu et al., 2012). These acids possessed notable inhibitory activity on the activated inflammatory cells, and which could be one of the main ingredients in supporting the anti-inflammatory activity of jujube (Yu et al., 2012). Besides, jujuboside and flavonoid in the fruit were also proposed to be active compounds, and which might responsible for anti-inflammatory effects (Goyal et al., 2011). Another animal study showed that jujube essential oil could inhibit the inflammatory responses of skin (Al-Reza et al., 2010).

In addition, jujube was reported to contain numerous minerals, e.g., iron and vitamin. About 0.48 mg iron and 69 mg vitamin C per 100 g of fresh fruit were reported (Li et al., 2007; USDA, 2012). Thus, the daily intake of jujube could increase our dietary iron and vitamin, as to prevent anemia due to deficiency of iron or vitamin C. Moreover, cAMP was found to have high abundance in jujube, and surprisingly this content was much higher than other horticultural fruits (Hanabusa et al., 1981). It is well accepted that increasing cAMP level can stimulate protein kinase A and, subsequently, which phosphorylates CREB (Argyrousi et al., 2020). Besides, jujube cAMP has been found to possess anti-melancholic effect in animal model of depression (Chi and Zhang, 2009). Thus, it is supposed that the cAMP in the jujube may account for its role on HIF (hypoxia inducible factor)-dependent EPO induction.

### Jujube on Erythropoiesis

**FIGURE 4:**
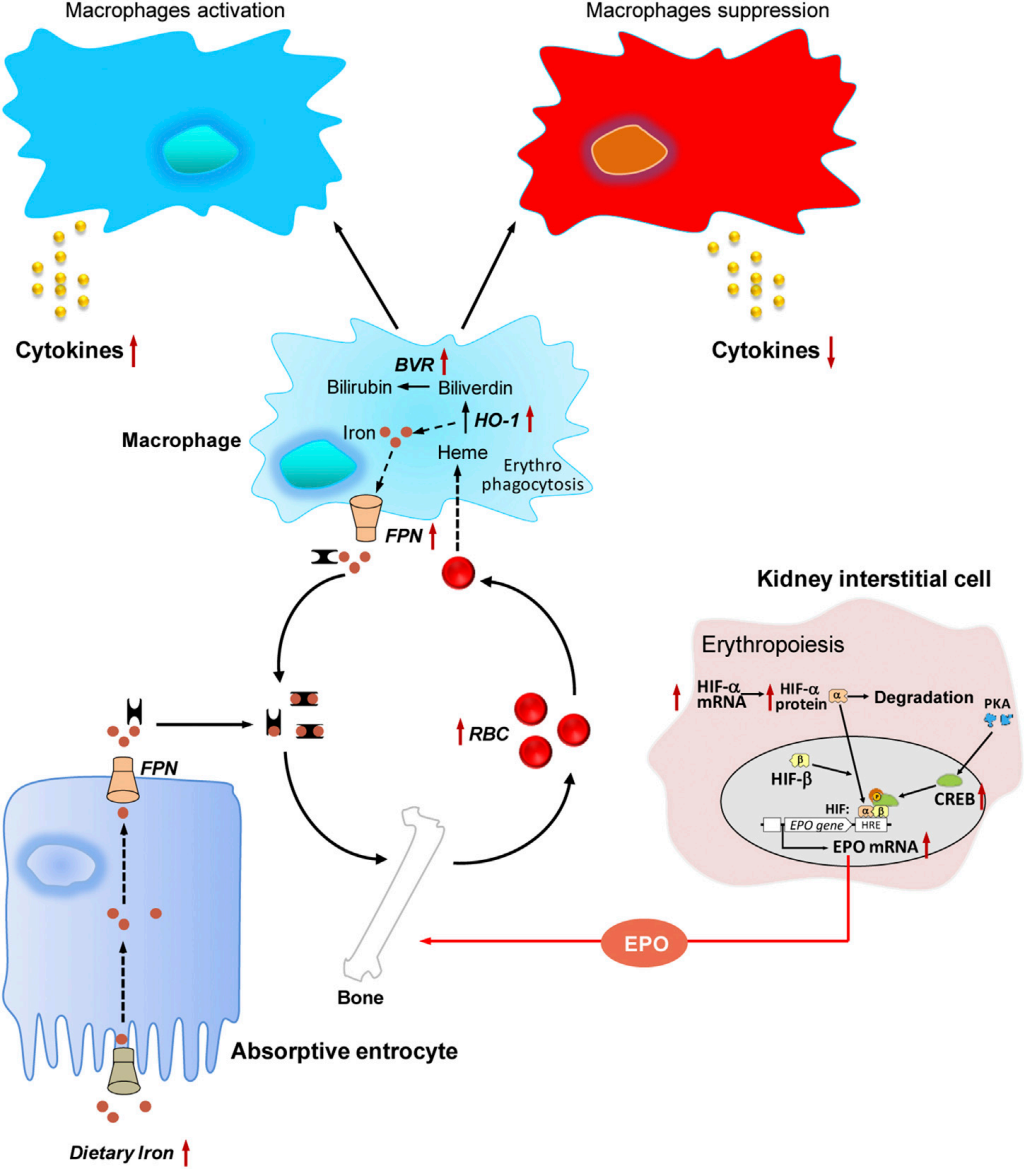
The hematopoietic functions of jujube. Jujube promotes erythropoiesis via activation of hypoxia inducible factor-induced erythropoietin, possesses potential capacity in recycling heme iron during erythrophagocytosis, exhibits bidirectional role in regulating immune response under different conditions, contains numerous minerals including iron. Red dots indicate iron, and yellow dots indicate cytokine. The arrow in red indicates the targets for jujube action. RBC, red blood cell; EPO, erythropoietin; HIF, hypoxia inducible factor; HRE, hypoxia response element; PKA, protein kinase A; CREB, cAMP response element-binding protein; FPN, ferroportin; BVR, biliverdin reductase; HO-1, heme oxygenase-1.

Erythropoiesis is considered to play critical roles in hematopoiesis, by which production of red blood cells (RBCs) is occurred. In this process, EPO, a RBC-specific hormone, is able to regulate erythropoiesis in bone marrow (Ascensao et al., 1991). EPO gene expression regulates primarily at the level of transcription, and which is further controlled by a number of transcriptional and post-transcriptional factors (Schuster et al., 1989; Goldberg et al., 1991). Failure to up regulate the circulating EPO under hypoxia thereafter leads to anemia (Jelkmann, 1992). Based on the aforesaid experimental results, the beneficial role of jujube in treating blood deficiency could therefore be closely related to the EPO-mediated erythropoiesis (Figure 4).

Jujube polysaccharide has been reported to improve hematological parameters in anemic animal models. Mice of blood deficiency model were induced by releasing blood and injection of cyclophosphamide. The levels of RBC, hemoglobin and hematocrit were decreased, and the level of platelet was increased in model mice, as compared to control group. Compared with model mice, the decreased levels of RBC, hemoglobin and hematocrit were reversed by treatment with jujube extract; while the increased level of platelet however was down regulated in jujube-treated mice (Xu et al., 2004). In another investigation, the activities of dietary jujube on the levels of RBC, hemoglobin and hematocrit were analyzed in colitis-associated colon cancer mice. These hematopoietic parameters in cancer mice were significantly decreased, as compared to control group; however, which were markedly increased in the jujube-treated mice (Periasamy et al., 2020), suggesting possible beneficial effects of this fruit on cancer patients suffering from anemia. Besides, the extract of jujube possessed the ability to stimulate the activity of ATPase, i.e., Na+-K+-ATPase, Ca^2+^-ATPase and Ca^2+^-Mg^2+^-ATPase, in erythrocyte, and therefore which was shown to promote bone marrow nuclear proliferation and to inhibit atrophy thymus and spleen in blood deficient animals (Miao et al., 2006; Miao et al., 2010). Moreover, the intake of jujube extract (100–400 mg/kg) was found to increase the level of EPO in blood circulation, which suggested that jujube might promote RBC level through up regulation of EPO production. In support of this notion, the applied jujube extract at concentrations of 0.75–3 mg/ml in cultured Hep3B cells for 48 h increased the expression of EPO transcription, and the increase was shown to be in a dose-dependent manner (Chen et al., 2014b). In parallel, the applied jujube extract was able to stimulate the protein expression of EPO, giving ∼50% increase of the total protein (Lam et al., 2016). The circulating EPO is produced by adult kidney cells; while kidney dysfunction contributes to inadequate amount of EPO production and renal anemia. In ibuprofen-induced nephrotoxicity rats, the intake of jujube extract (500 mg/kg) improved kidney function by declining the levels of creatinine and urea, and this treatment could prevent histopathological damages of kidney (Awad et al., 2014). On the other hand, the intake of Jian-Pi-Yi-Shen, a Chinese herbal decoction comprising of Astragali Radix, Atractylodis Macrocephalae Rhizoma, Dioscoreae Rhizoma, Cistanches Herba, and other four herbs, was able to improve renal function and kidney injury in anemia rats suffering from chronic kidney disease (Chen et al., 2019). Jian-Pi-Yi-Shen improved the hematological parameters and stimulated EPO production (Chen et al., 2019). Massive EPO-producing cells are identified in the renal interstitium. The occurrence of renal interstitial fibrosis is accompanied by decrease of fibroblasts, which impairs the production of EPO. Moreover, Jian-Pi-Yi-Shen was believed to ameliorate renal interstitial fibrosis, and the renal recovery might be related to improvement of EPO production. Thus, the function of jujube in promoting EPO expression in renal anemia patients was in line to that of Jian-Pi-Yi-Shen (Chen et al., 2019). This assumption requires further studies as to confirm the ability of jujube to prevent/treat renal anemia through regulation of EPO production.

The promoter of *EPO* gene contains HRE, and thus the activation of hypoxia-mediated signaling pathway is leading to activation of EPO expression (Post and Van Meir, 2001). Cultured Hep3B cells were transfected with HRE promoter fragment (i.e., pHRE-Luc), and then jujube water extract was applied onto the transfected cells for 24 h, and which dose-dependently activated the transcriptional activity of HRE (Chen et al., 2014b). To account for the possible mechanism of HIF signaling in jujube-induced HRE activation, the expression of HIF-1α was determined. The jujube extract at various concentrations (0.75–3 mg/ml) was applied onto cultured Hep3B cells for 6 h, and then total RNA was harvested from the cultures for PCR analysis. Jujube extract stimulated the expression of HIF-1α mRNA, and the induced expression was demonstrated to be in a dose-dependent manner, having the highest effect by ∼80% of increase. In parallel, the protein level of HIF-1α in the cultures turned to increase after 2 h, and subsequently HIF-1α protein was induced by ∼150% at 6 h after the treatment (Chen et al., 2014b). These results support the effect of jujube on HIF-1α expression in both mRNA and protein levels. In addition, Xu et al. (2014) reported that CREB-binding protein was required for HIF-α acetylation and efficient HIF-mediated EPO production during hypoxic stress. In consistent with this, Chen et al. (2014c) found that jujube extract (2 mg/ml) induced CREB phosphorylation in cultured cells, and this effect was fully blocked by H89, a cyclic AMP-dependent protein kinase A inhibitor. These results indicate that CREB-binding protein/HIF signaling can be involved in jujube-induced EPO production.

### Jujube on Erythrophagocytosis

Erythrophagocytosis is a process, where the senescent RBCs are phagocytosed by macrophage (Klei et al., 2017). Within the macrophage, the senescent RBC undergoes hemolysis, and the components, such as heme iron, are being recycled. The reused iron will be carried back to bone marrow for erythropoiesis (Gottlieb et al., 2012). Thus, the disorders in iron recycling can result in anemia (Batchelor et al., 2020). Here, the potential effects of jujube on erythrophagocytosis were summarized (Figure 4).

Heme oxygenase-1 (HO-1) has a vital role in metabolizing heme to biliverdin, carbon monoxide and free iron. Biliverdin is immediately conversed to bilirubin, as catalyzed by biliverdin reductase containing two isozymes, i.e., biliverdin reductase A and B. Free iron is released to blood circulation by ferroportin and further carried to bone marrow (Kovtunovych et al., 2010). Therefore, HO-1, biliverdin reductase and ferropotin are considered as the main target enzymes in determining the iron recycling in macrophages. Jujube extract at different concentration (0–3.0 mg/ml) was applied onto cultured macrophages for 24 h. The applied jujube extract stimulated the mRNA expressions of HO-1, biliverdin reductase A and B, and ferropotin in dose-dependent manners, giving the highest response by ∼2.0, 2.0, 3.0, and 4.0 folds, respectively (Chen J. et al., 2016). In good agreement with this finding, Yang et al. (2016) reported that the intake of jujube extract showed an improvement in iron deficiency anemia rats. In parallel, the extract of jujube significantly increased serum iron, iron saturation, total iron binding capacity in anemia rats, indicating the supply of circulation iron for erythropoiesis. Nuclear factor (erythroid-derived 2)-like 2 (Nrf2), a transcription factor, was found to regulate HO-1 expression (Motohashi and Yamamoto, 2004). Nrf2 directly binds to anti-oxidant response element (ARE) in the promoter region of *HO-1* gene resulting in the transcription. In pARE-Luc-expressed cells treated with jujube water extract, the luciferase assay was activated in a dose-dependent manner (Chen J. et al., 2016). The activation by two folds was confirmed under application of jujube extract at 3.0 mg/ml (Chen J. et al., 2016). This result suggests the involvement of Nrf2/HO-1 signaling in jujube-treated cells. In support of this notion, Almeer et al. (2018) revealed the effects of jujube extract on gene expressions of Nrf2 and HO-1 in colitis rats, as induced by treating intrarectally with acetic acid. Compared with model group, the pre-treatment with jujube extract at different doses (100, 200, and 400 mg/kg/day) in colon of rats for 5 days by oral gavage significantly induced mRNA expressions of Nrf2 and HO-1, giving the highest response by seven and two folds, respectively (Almeer et al., 2018). These studies however need further confirmation as no observation has been found in a physiology model of iron recycling. For instance, a cellular model of erythrophagocytosis using artificially-aged RBCs and macrophages may be designed to investigate mRNA and protein expressions of target enzymes relating to iron recycling.

### Jujube on Immune Functions

The immune response is impaired in anemia condition. Here, the immune-modulatory properties of jujube under different scenarios are summarized (Figure 4). The intake of jujube extracts at concentrations of 150 and 250 mg/kg/day significantly stimulated thymus and spleen indices in mice, which indicated obviously strengthening the non-specific immunity in jujube-treated mice (Li et al., 2011). Furthermore, the authors described that jujube at various concentrations (30–200 μg/ml) showed a significant dose-dependent promotion of splenocyte proliferation, with the highest response at ∼100% increase. The effect of jujube extract on anti-complementary activity was also reported. Jujube extracts (25 and 125 μg/ml) exhibited ability to interact with the complement cascade (Li et al., 2011). This finding indicates the activation of innate immune system in jujube-treated cells. In support of this notion, the water-soluble polysaccharide isolated from jujube stimulated proliferation of lymphocyte. In particular, the application of jujube polysaccharide at various concentrations (10–100 μg/ml) onto cultured lymphocytes for 3 days demonstrated an enlarging cell volume and an increasing cell number (Zhao et al., 2008). Moreover, jujube extracts at different concentrations of 0–3 mg/ml were applied onto cultured RAW 264.7 cells for 24 h. The extract dose-dependently stimulated the expressions of interleukin (IL)-1β, IL-6 and tumor necrosis factor (TNF)-α, and the highest effect was induced at ∼7-fold, ∼9-fold, and 4-fold, respectively (Chen et al., 2014a).

The hydroalcoholic extract of jujube at 200–400 mg/kg was applied onto acute and chronic rat models of inflammation. The study revealed that jujube extract markedly declined granuloma tissue formation, as compared with model rat. Serum nitrite/nitrate level was notably up regulated in inflammatory rat; while pre-treatment with jujube significantly reduced the increased level of nitrite/nitrate (Goyal et al., 2011). These findings suggest the anti-inflammatory effects of jujube. In line with this, the applied jujube extract at 100–500 μg/ml inhibited nitric oxide production and splenocyte proliferation on the inflammatory activated cells (Yu et al., 2012). The excessive induction of pro-inflammatory cytokines, i.e., IL-1β and IL-6, also contributes to chronic inflammation. In the lipopolysaccharide (LPS)-induced macrophages, the pre-treatment with jujube water extract repressed the expressions of IL-1β and IL-6 (Chen et al., 2014a). Besides, the triterpene acid fraction of jujube at a dose higher than 10 μg/ml was able to inhibit the production TNF-α (Yu et al., 2012). NF-κB is one of main transcription factors that has vital role in controlling pro-inflammatory cytokine production (Du et al., 2013). The treatment with jujube extract slightly inhibited NF-κB activity. On the other hand, the pre-treatment with jujube water extract (0–3 mg/ml) for 3 h in cultured macrophages, before the addition of LPS at 1 μg/ml for 24 h, dose-dependently repressed the activation of NF-κB activity. The reduction at 60% was observed under the pre-treatment of extract at 3 mg/ml (Chen et al., 2014a). In parallel, the anti-inflammatory property of jujube was shown in colitis-associated colon cancer mice (Periasamy et al., 2020). In this study, mice were injected with azoxymethane followed by three cycles of dextran sulfate sodium, as to induce colitis-associated colon, before the intake of jujube extract for 70 days. The results showed that dietary jujube intake could attenuate inflammation in model mice. Moreover, the extract of jujube markedly inhibited the protein expressions of IL-6, NF-κB, JAK1, and STAT3 in colon tissues, as compared with model group. This result suggests that jujube can suppress the stimulation of NF-κB/IL-6/JAK1/STAT3 signaling (Periasamy et al., 2020).

### Jujube in Blood Circulation

Blood is circulating within the blood vessels, and the effects of jujube in blood circulation are summarized here. The pre-incubation of jujube extract (30, 100, and 300 mg/ml) for 5 min at 37°C in platelet-rich plasma was reported to suppress collagen (2 mg/ml)-, thrombin (0.4 U/ml)-, and arachidonic acid (100 mM)-induced aggregation of platelets (Seo et al., 2013). Jujuboside B from jujube markedly inhibited platelet aggregation, and which was considered as one of the active ingredients in processing anti-platelet effect (Seo et al., 2013). In animal model, angiotensin II was intravenously injected into rats to induce acute hypertension, which characterized by cardiovascular parameters, i.e., notably elevated systolic blood pressure and mean arterial pressure, as well as the decline of heart rate (HR) compared with control group. Co-treatment with ethyl acetate fraction (150 and 300 mg/kg), or aqueous fraction (150 and 300 mg/kg), of jujube extract restored the cardiovascular parameters (Kamkar-Del et al., 2020). In line with this notion, Mohebbati et al. (2018) reported protective effect of jujube extract on hypertensive rats. The rats were treated with hydroalcoholic extracts of jujube at various concentrations (from 100 to 400 mg/kg) for four weeks, and then L-NAME (10 mg/kg) was injected intravenously into rats to induce hypertension. The results showed that jujube extract attenuated blood pressure and mean arterial pressure in L-NAME-induced hypertensive rats (Mohebbati et al., 2018). Betulinic acid, found in Zizyphi Spinosi Semen and jujube, possessed combined properties of inducing endothelial nitric oxide synthase and decreasing nicotinamide adenine dinucleotide phosphate oxidase. In human endothelial cells treated with betulinic acid, the endothelial nitric oxide synthase expression and nitric oxide production were robustly increased (Steinkamp-Fenske et al., 2007). In addition, a triple-masked randomized controlled clinical trial revealed that jujube was well tolerated in general, and which might possess potential beneficial roles on serum lipid profile (Sabzghabaee et al., 2013).

### Jujube in Cocktail Recipes

Jujube is not only consumed as daily food, but also prescribed as a tonic TCM for blood nourishment in a formulated decoction. Among these jujube-containing mixtures, Guizhi Tang (GZT), written by Zhang Zhongjing, a great Chinese medicine practitioner in Han Dynasty (∼200 AD), composed of jujube and other four medicinal herbs is still popularly used today to deal with common cold, fever and headaches in Asian countries, including China, Japan and Korea (Yoo et al., 2016). GZT belongs to exterior-releasing formula that is able to dispel pathogenic factors from superficies of body, and the effect of jujube within this formula is believed to tonify “Qi” and to replenish “Blood” of the body resulting from corresponding pathological changes. Yoo et al. (2016) reported that application of GZT extract in cultured RAW264.7 macrophages showed anti-inflammatory activity, which involved in blocking ERK and NF-κB signaling pathways. The cells were pre-treated with GZT (31.25–1,000 μg/ml) for 4 h prior to application of LPS for an additional 20 h. The treatment with GZT extract enhanced the expression of HO-1 and significantly inhibited pro-inflammatory cytokines, e.g., TNF-α and IL-6, in LPS-induced macrophages. Besides, GZT extract prevented ERK phosphorylation and NF-κB translocation in LPS-treated macrophages (Yoo et al., 2016). In another experiment, Lam et al. (2016) investigated the inductive roles of GZT on EPO expression in cultures. Cultured Hep3B cells were treated with GZT extracts (0.5–4.0 mg/ml) for 24 h. Applied GZT was able to stimulate the mRNA and protein expressions of EPO. In addition, GZT stimulated the transcriptional activity of HRE in a dose-dependent manner (Lam et al., 2016). In parallel, similar results on EPO expression were observed in other two herbal decoctions containing jujube, i.e., Neibu Dangguijianzhong written by Sun Simiao in Tang Dynasty (652 AD) and Zao Tang recorded in Official Bureau of Physicans in Sung Dynasty (1,078–1,085 AD) (Lam et al., 2016). In Zhigancao Tang, jujube combining with ginseng was used to tonify the “Qi.” Besides, jujube was proposed to regulate the relationship between protective and nutritive “Qi.” Nevertheless, jujube showed similar function with other herbal formulae in treating “Qi” and “Blood” deficiency, e.g., Renshen Yangying Tang, Bazhen Tang and Xiangbei Yangying Tang.

In addition to formulated decoction, jujube is also commonly supplemented with other foods to achieve health benefits. Jeong and Kim (2019) investigated the effect of jujube and chokeberry diet in high-fat and high-fructose diet-induced dyslipidemia in animal studies. Jujube (0.5%) and chokeberry powder (0.5%) were mixed to animal diet. After 10 weeks of dietary treatment, jujube and chokeberry significantly ameliorated high-fat and high-fructose diet-induced dyslipidemia and improved insulin resistance (Jeong and Kim, 2019). In support of this finding, the consumption of mixed jujube, almond and rice in healthy human showed a significantly lower glucose level, as compared to those with rice as reference (Zhu et al., 2018).

### Future Opportunities

Herbal cuisine is a practice in achieving the therapeutic functions by using natural herbs, especially the edible and medicinal dual-purpose herbs as materials during cooking processes. Jujube has been considered as a favorite fruit in daily life for its health properties spanning thousands of years. In practice of herbal cuisine, jujube is one of common materials that is considered as boosting or nourishing type of food. It can be taken into decoction for daily consumption, or which can be taken together with other foods to prepare delicious soup. According to the aforesaid cellular and animal findings, jujube has a promising potential in developing medicinal food and supplement for prevention against anemia, cancer, inflammation and iron/vitamin deficiency.

Jujube-containing herbal decoctions are routinely recorded in *Jingui Yaolue* by Zhang Zhongjing, which are prescribed to address various ailments. One-sixth of prescriptions described in this ancient classic book contain jujube, in which jujube is commonly served as assistant or courier medicinal herb with a herbal formulated decoction, according to the theory of TCM. The intake of jujube is believed to increase blood supply to the spleen meridian that further improves nutrient uptake and strengthens the immune system. Clinically, several controlled trials have revealed that jujube is a safe and effective herb for human consumption (Naftali et al., 2008; Ebrahimimd et al., 2011; Sabzghabaee et al., 2013); however, there is currently no human study on the blood deficiency effects. In addition, there are no known toxicity and drug interaction being reported clinically for consumption of jujube.

Apart from the medicinal application, fresh immature jujubes are widely consumed as fruits, and the dried fruits are also eaten as a snack, or with tea. In China, jujube has been made into a wide range of products, i.e., juice, vinegar and wine. In southern part of India, jujube is mixed with tamarind, jaggery, salt and chilies, and then pounded into cakes. In Lebanon and Persia, jujube is used as digestive aid being consumed with the desserts. In Morocco, the honey obtained from jujube extract is believed to be beneficial to sore throats (Lim, 2013). Additional of natural herbal extracts to dairy products has increasingly popular because of their health benefits. Feng et al. (2019) demonstrated a new goat dairy product adding of jujube pulp as ingredients with satisfactory nutritional quality and sensory property, which provided alternative approach in developing a unique goat dairy product with high nutritional properties. The water extract of immature jujube extract showed better activity in stimulating transcriptional activity of HRE than that of mature jujube (Chen et al., 2015). These findings indicate the maturity of jujube should therefore be taken into consideration when it is being prepared for health food supplements.

In folk medicine, three pieces of jujube are recommended to be consumed daily, about 15 g of dried weight in total. The content of benefit ingredients within jujube could be changed robustly between fresh jujubes and dried ones (Guo et al., 2015; USDA, 2012; Chen et al., 2013). The dietary nutrient facts of main ingredients in fresh and dried jujubes are summarized in Supplementary Table S1, which provides an appropriate recommendation for selection of different forms of jujube for certain health benefits. In addition, it has been reported that the content of bio-active ingredients, including nucleotide, flavonoid and polysaccharide, varied among different jujube cultivars (Chen et al., 2013), as indicated in Supplementary Table S2. Jujubes from Shanxi, Shaanxi, Hebei, Xinjiang, Shandong, Ningxia provinces of China had higher chemical amounts, which might contribute better biological functions and could be a good choice of selection.

## Author Contributions

JC and KT: Concept, design, literature search and manuscript review. JC: acquisition of data, drafting the manuscript. All authors have read and approved the manuscript.

## Funding

This work is supported by Natural Science Foundation of Guangdong Province (2018A030313305), Natural Science Foundation of China (81804052), Shenzhen Science and Technology Plan Project (JSGG20191129102216637 and ZDSYS201606081515458), Traditional Chinese Medicine Bureau of Guangdong Province (20201320) to JC. Shenzhen Science and Technology Innovation Committee (ZDSYS201707281432317; JCYJ20170413173747440; JCYJ20180306174903174), China Post-doctoral Science Foundation (2019M653087), Zhongshan Municipal Bureau of Science and Technology (ZSST20SC03); Guangzhou Science and Technology Committee Research Grant (GZSTI16SC02; GZSTI17SC02); Hong Kong RGC Theme-based Research Scheme (T13-605/18-W); Hong Kong Innovation Technology Fund (UIM/340, UIM/385, ITS/500/18FP; TCPD/17-9); TUYF19SC02, PD18SC01 and HMRF18SC06 to KT.

## Conflict of Interest

The authors declare that the research was conducted in the absence of any commercial or financial relationships that could be construed as a potential conflict of interest.
