# Supplementary material for: A Review of Edible Jujube, the Ziziphus jujuba Fruit: A Heath Food Supplement for Anemia Prevalence
Source: Front Pharmacol. 2020 Nov 26;11:593655. doi: 10.3389/fphar.2020.593655 (PMC7726020; doi:10.3389/fphar.2020.593655)
Supplement: Supplementary file 1 [file DataSheet1_v1.docx]

**Table S1 Nutrient composition of fresh jujubes and dried jujubes**

| **Nutrient** | **Unit** | **Value per 100 g** | | **Reference** |
| --- | --- | --- | --- | --- |
|  |  | Fresh ^a^ | Dried |  |
| Energy | g | 79 | 287 | USDA, 2012 |
| Iron, Fe | mg | 0.48 | 1.80 | USDA, 2012 |
| Vitamin C | mg | 69.0 | 13.0 | USDA, 2012 |
| Sugars ^b^ | g | 6.94 | 7.20 | Guo et al., 2015 |
| Nucleotides | mg | 49.50 | 71.98 | Chen et al, 2013 |
| Flavonoids | mg | 73.28 | 48.62 | Chen et al, 2013 |
| Triterpenic acids | mg | 222.33 | 343.43 | Guo et al., 2015 |
| Polysaccharides | g | 2.80 | 3.30 | Chen et al, 2013 |

^a^The water content of fresh and dried jujubes is about 70% and 20%.

^b^The amount of sugar is expressed as sum of sucrose, glucose and fructose.

**Table S2 The contents of ingredients in different jujubes.**

|  | Uracil ^b^ | Cytidine | Uridine | cGMP | Xanthine | Hypoxanthine | Guanine | cAMP | Adenine | (-)-Catechin | Procyanidin B2 | (-)-Epicatechin | Quercetin 3-O-galactoside | Quercetin 3-O-rutinoside | Quercetin 3-O-β-D-glucoside | Kaempferol 3-O-rutinoside | Polysaccharides |
| --- | --- | --- | --- | --- | --- | --- | --- | --- | --- | --- | --- | --- | --- | --- | --- | --- | --- |
| A^a^ | 3.79 | 26.93 | 194.51 | 108.10 | 66.50 | 25.10 | 12.24 | 196.24 | 58.29 | 4.02 | 7.48 | 3.07 | 0.24 | 25.50 | 0.68 | 0.70 | 34 |
| B | 13.06 | 36.21 | 168.66 | 33.10 | 5.28 | 1.66 | 4.11 | 49.17 | 56.11 | 20.53 | 39.01 | 26.26 | 0.60 | 78.61 | 1.40 | 1.12 | 38 |
| C | 6.87 | 14.74 | 96.54 | 73.36 | 1.83 | 5.76 | 5.00 | 151.03 | 33.83 | 12.97 | 9.93 | 11.20 | 0.21 | 40.01 | 0.33 | 2.09 | 42 |
| D | 14.94 | 17.52 | 137.30 | 35.98 | 2.23 | 10.20 | 5.33 | 106.94 | 22.32 | 3.02 | 0.62 | 1.76 | 0.17 | 59.29 | 0.39 | 2.22 | 43 |
| E | 10.94 | 55.88 | 113.82 | 85.06 | 47.75 | 4.31 | 5.66 | 171.58 | 44.29 | 12.03 | 15.52 | 9.71 | 0.14 | 66.90 | 0.85 | 1.05 | 37 |
| F | 6.41 | 23.77 | 161.84 | 164.58 | 35.71 | 4.52 | 7.23 | 532.79 | 58.08 | 12.42 | 8.86 | 9.37 | 0.15 | 48.26 | 0.38 | 1.61 | 32 |
| G | 5.14 | 18.06 | 112.08 | 66.04 | 40.87 | 3.47 | 2.86 | 138.88 | 47.11 | 13.18 | 31.23 | 19.05 | 0.35 | 57.04 | 0.69 | 0.42 | 29 |
| H | 11.77 | 20.85 | 102.34 | 42.21 | 18.79 | 6.20 | 3.39 | 88.24 | 44.29 | 10.61 | 11.88 | 5.82 | 0.41 | 48.27 | 0.95 | 0.44 | 34 |
| I | 7.83 | 24.82 | 173.56 | 80.81 | 6.41 | 2.54 | 7.25 | 145.59 | 52.07 | 28.00 | 66.69 | 32.98 | 0.43 | 58.78 | 0.99 | 0.31 | 27 |
| J | 4.84 | 25.48 | 134.32 | 80.44 | 4.85 | 6.94 | 2.70 | 168.42 | 50.59 | 40.97 | 216.67 | 51.82 | 0.62 | 78.73 | 1.94 | 1.49 | 42 |
| K | 6.21 | 26.54 | 134.77 | 64.36 | 14.58 | 9.35 | 2.19 | 243.71 | 58.81 | 4.83 | 2.77 | 3.08 | 0.09 | 13.46 | 0.33 | 0.97 | 31 |
| L | 8.09 | 25.43 | 108.04 | 126.21 | 13.57 | 3.62 | 4.06 | 375.15 | 56.35 | 8.61 | 14.68 | 7.63 | 0.24 | 32.48 | 0.83 | 1.08 | 38 |
| M | 10.62 | 62.69 | 121.09 | 107.34 | 25.03 | 10.36 | 4.73 | 195.50 | 47.64 | 7.20 | 8.24 | 6.00 | 0.24 | 38.60 | 0.75 | 1.60 | 42 |
| N | 5.24 | 38.47 | 122.80 | 92.79 | 21.71 | 2.99 | 5.60 | 187.67 | 47.37 | 6.07 | 2.65 | 4.77 | 0.13 | 24.81 | 0.23 | 2.19 | 44 |
| O | 13.21 | 25.85 | 187.47 | 158.03 | 2.74 | 12.83 | 4.56 | 593.83 | 77.06 | 6.25 | 4.19 | 5.13 | 0.19 | 39.38 | 0.64 | 7.45 | 29 |
| P | 12.05 | 17.77 | 179.77 | 61.47 | 29.79 | 2.33 | 11.85 | 124.90 | 48.54 | 6.63 | 4.41 | 8.45 | 0.03 | 17.05 | 0.08 | 3.98 | 28 |
| Q | 4.56 | 33.73 | 151.09 | 104.79 | 26.92 | 5.24 | 3.95 | 209.95 | 51.53 | 11.97 | 13.69 | 10.71 | 0.07 | 18.91 | 0.18 | 0.89 | 35 |
| R | 6.98 | 16.91 | 165.80 | 143.82 | 1.98 | 11.52 | 3.84 | 501.38 | 63.70 | 6.68 | 4.57 | 5.21 | 0.19 | 37.48 | 0.77 | 4.81 | 34 |
| S | 13.81 | 44.74 | 180.84 | 67.37 | 4.17 | 13.24 | 2.41 | 171.35 | 31.72 | 3.00 | 2.82 | 3.13 | 0.07 | 5.58 | 0.10 | 0.47 | 49 |
| T | 16.16 | 19.86 | 145.84 | 60.80 | 2.02 | 25.41 | 11.08 | 117.55 | 42.33 | 4.78 | 2.63 | 5.73 | 0.04 | 14.25 | 0.13 | 5.10 | 33 |
| U | 11.71 | 23.34 | 167.56 | 130.66 | 3.03 | 8.87 | 1.66 | 519.12 | 78.44 | 5.26 | 4.04 | 4.61 | 0.12 | 18.76 | 0.30 | 1.77 | 30 |
| V | 19.14 | 70.96 | 161.38 | 111.02 | 9.21 | 2.41 | 4.10 | 348.70 | 50.46 | 8.18 | 7.84 | 6.11 | 0.25 | 37.42 | 0.84 | 0.66 | 37 |
| W | 5.87 | 53.96 | 163.52 | 110.43 | 6.43 | 1.67 | 5.43 | 307.66 | 72.47 | 6.38 | 8.25 | 5.45 | 0.29 | 27.68 | 0.91 | 0.69 | 45 |
| X | 7.32 | 58.51 | 166.30 | 96.92 | 23.69 | 4.43 | 6.50 | 253.08 | 53.49 | 14.54 | 22.24 | 12.18 | 0.22 | 35.27 | 0.77 | 0.38 | 29 |

^a^The notation of A to X are corresponding to jujubes from different regions of China, and the detail information of each sample can refer to Chen et al., 2013.

#### ^b^ Values are expressed in μg/g of dried weight except polysaccharides are in mg/g.
